# Pyogenic Flexor Tenosynovitis by Point-of-care Ultrasound in the Emergency Department

**DOI:** 10.5811/cpcem.2018.3.37415

**Published:** 2018-07-09

**Authors:** Daniel Hubbard, Scott Joing, Steven W. Smith

**Affiliations:** * Oregon Health & Science University, Department of Emergency Medicine, Portland, Oregon; † Hennepin County Medical Center, Department of Emergency Medicine, Minneapolis, Minnesota

## Abstract

**Introduction:**

Pyogenic flexor tenosynovitis (PFT) is difficult to diagnose on clinical grounds alone as many patients requiring an operation do not have all four of Kanavel’s signs. Previous studies have shown that hypoechoic fluid surrounding the flexor tendon on ultrasound is associated with this diagnosis. We sought to determine if emergency physicians (EPs) could recognize this finding in patients with suspected flexor tenosynovitis using point-of-care ultrasound (POCUS).

**Methods:**

We present a retrospective case series of seven patients suspected of PFT who had hypoechoic fluid surrounding the tendon on POCUS performed by the treating EP. We report on the patient characteristics, history of trauma by puncture wound, number of Kanavel’s signs, treatment course, and operative findings.

**Results:**

We identified seven patients suspected to have flexor tenosynovitis by the emergency department attending physician who had anechoic or hypoechoic fluid surrounding the flexor tendon on real-time POCUS examination. Patients ranged in age from 16 – 51 years. All were male. All patients had at least two of Kanavel’s signs on examination. Five of seven (71%) patients had history of recent trauma to the affected hand. Four of seven (57%) were managed in the operating room. One of seven (14%) had incision and drainage at the bedside, and the remaining two (28%) were managed non-operatively and successfully with antibiotics alone.

**Conclusion:**

Our study demonstrates that EPs can recognize the finding of hypoechoic or anechoic fluid surrounding the flexor tendon on POCUS.

## INTRODUCTION

Dr. Allen B. Kanavel’s textbook, *Infections of the Hand. A Guide to the Surgical Treatment of Acute and Chronic Suppurative Processes in the Fingers, Hand, and Forearm,* was first published in 1912. The now-eponymous Kanavel’s signs are as follows: 1) fusiform swelling; 2) finger held in partially flexed position; 3) pain on palpation of the flexor tendon; and 4) pain on passive stretch of the flexor tendon.^1^ Since the first publication of these signs more than 100 years ago there have been no prospective data to determine their sensitivity and specificity. Retrospective series have come to different conclusions regarding the significance of the different signs.

Dailiana et al. in 2008 found that all four of Kanavel’s signs were present in only 22/41 (54%) patients taken to the operating room (OR) with acute pyogenic flexor tenosynovitis (PFT); they noted, however, that all 41 patients had tenderness along the tendon and pain on passive stretch.^2^ Pang et al., in a series of 75 patients published in 2007, noted that only 72% of patients had pain on passive stretch and just 64% had tenderness along the tendon. This study noted that fusiform swelling was the most common finding (97%).^3^ Thus, healthcare providers are still left with diagnostic uncertainty in patients presenting to the emergency department (ED) with painful, swollen fingers because physical examination criteria alone are not sufficient to identify patients requiring surgery.

Since at least the 1980s ultrasound (US) has been employed to attempt to differentiate between flexor tenosynovitis and other infections of the hand.^4^,^5^ These previous studies found that hypoechoic or anechoic fluid surrounding the flexor tendon is associated with purulent discharge at time of surgery and thus a useful adjunct in the diagnosis of PFT. US is particularly useful because the normal finger does not have appreciable synovial fluid on US images.^6^ Accordingly, presence of fluid is abnormal and, in the setting of clinical infection, suggests tenosynovitis. There have been at least three individual case reports from the emergency medicine (EM) point-of-care ultrasound (POCUS) literature in which a finding of fluid surrounding the flexor tendon in a patient presenting with multiple Kanavel signs was determined to be purulent at time of surgery.^7^–^9^ To date there have been no case series from the EM literature on this topic.

The objective of this study was to determine if emergency physicians (EP) could recognize this finding on patients with suspected flexor tenosynovitis by POCUS. We present here a case series of seven patients presenting to the ED with at least two of Kanavel’s signs on exam and fluid seen on POCUS performed by an EP.

## MATERIALS AND METHODS

This was a retrospective case series of patients presenting to our urban, academic emergency department (ED) over an eight-year time period. Our ED attending physicians have been using POCUS since the 1980s, and all are credentialed in the use of bedside US in our institution. Real-time sonography was performed in each patient using a high-frequency linear probe and either a water bath or gel mattress. All patients were determined to have anechoic or hypoechoic fluid surrounding a flexor tendon on POCUS performed by the ED attending. We identified cases by performing a search of the departmental POCUS image archive system for a diagnosis of “tenosynovitis.” The electronic medical record was then reviewed for information regarding history of presenting illness, exam, additional diagnostics, specialist consultations, treatments, operative reports, hospital course, and follow-up (if available). All patients described here were found to have the finding of fluid surrounding the flexor tendon by POCUS by the ED attending. We only included cases if the consulting hand surgeon made a diagnosis of flexor tenosynovitis, with the exception of one case in which the final diagnosis by the consultant was “possible flexor tenosynovitis.” The institutional review board (IRB) at our hospital does not require IRB approval for retrospective case series using deidentified information.

**CPC-EM Capsule**

What do we already know about this clinical entity?

For over 100 years the diagnostic criteria for pyogenic flexor tenosynovitis have been the presence of Kanavel’s four cardinal signs on physical exam.

What makes this presentation of disease reportable?

This series demonstrates that ultrasound can be used to detect purulence within the tendon sheath and can do so in cases with an incomplete number of Kanavel’s signs.

What is the major learning point?

Point-of-care ultrasound can be used to identify purulence in the tendon sheath.

How might this improve emergency medicine practice?

Recognition of flexor tenosynovitis by visualization of fluid in the tendon sheath in patients without all four Kanavel’s signs might lead to improved patient outcomes.

## RESULTS

We identified seven patients who had anechoic or hypoechoic fluid surrounding the flexor tendon on real-time POCUS examination and were suspected to have flexor tenosynovitis by the ED attending Patients ranged in age from 16 – 51 years, and all were male. All patients had at least two of Kanavel’s signs on ED examination. Five of seven (71%) had history of recent trauma to the affected hand. Four of seven (57%) were managed in the OR. One of seven (14%) had incision and drainage (I&D) at the bedside, and the remaining two (28%) were managed non-operatively and successfully with antibiotics alone. See the table for summary of patient characteristics and key POCUS images.

## DISCUSSION

The currently available data on the association of fluid with purulent drainage at operation are scant. To our knowledge only two case series on the use of US in the diagnosis of acute PFT have been published, both in the 1980s by an identical author group.^4^,^5^ In these two case series of patients with suspected acute hand infections, anechoic or hypoechoic fluid surrounding the tendons was associated with PFT. The first series by Jeffrey et al.^4^ in 1987 detailed a total of seven patients suspected of having purulent flexor tenosynovitis who underwent US. Six patients had hypoechoic fluid areas surrounding the flexor tendon. Five of six patients with hypoechoic fluid surrounding the tendon on US had a final diagnosis of acute bacterial tenosynovitis with purulent drainage at surgery. Fluid around the tendon in the remaining patient was attributed to a viral inflammatory etiology. The patient in the study who did not have fluid surrounding the tendon on US but was found to have purulent tenosynovitis at surgery was noted to have 25% enlargement of the tendon on the affected side compared to the normal contralateral side. In fact, all six patients with bacterial flexor tenosynovitis had at least 25% enlargement of the affected tendon.^4^ In our present cases series, we did not assess tendon diameter.

The group’s second series,^5^ consisting of 18 patients and published in 1989, had somewhat mixed results. All 18 patients had >25% increase in tendon diameter by comparison to normal contralateral tendon. Twelve patients were taken to surgery, 11 of whom had the sonographic finding of fluid surrounding the tendon. However, purulence was found in only eight of these 11 US-positive cases. No explanation for the discrepancy between the sonographic and operative findings was offered. The authors do not report the time between image acquisition and surgery. Also of note, of the six remaining patients who were medically managed, one had fluid surrounding the tendon.

These series were published in 1987 and 1989 respectively, in an era in which US images were of inferior quality to today’s US images. It is possible that artifacts were falsely interpreted as fluid in the negative operative cases. It is also possible that medical management, including the administration of parenteral antibiotics, allowed for the arrest of the suppurative process and reabsorption of fluid prior to operative exploration. It is possible that contemporary machines may also increase the sensitivity of diagnosing PFT, but may also be detecting early cases in which surgical drainage is not necessary to achieve a cure, and antibiotics alone may be sufficient.

Our findings are notable in that despite the presence of fluid on US and multiple Kanavel’s signs, not all patients required operative treatment. It is unclear whether this was due to the absence of tenosynovitis or to early diagnosis with successful resolution with antibiotics alone. It is uncertain what role the US played in the diagnosis, but all who were evaluated for the possibility of tenosynovitis were found to have abnormal fluid. Patients with only two of Kanavel’s signs might have gone undiagnosed without US. It is possible that US identifies patients with tenosynovitis with such high sensitivity that it could lead to over-triage to the OR. On the other hand, its sensitivity could lead to early diagnosis, appropriate antibiotic therapy, and beneficial avoidance of operative treatment. We did not report on all cases of tenosynovitis. It is possible that not all cases would have fluid and that a negative US could lead to a false dismissal of the diagnosis or even influence management away from surgery when surgery is actually necessary. Only a prospective trial could answer these questions. A finding of fluid in the sheath is also consistent with non-pyogenic processes; it is well documented that various rheumatologic conditions are also associated with fluid surrounding the flexor tendons.^10^,^11^

All patients selected for our retrospective analysis were included because they presented with a suspected acute hand infection and were found to have fluid around the flexor tendon at the time of ED evaluation. A conclusion from the previously published data reviewed is that the finding of fluid around the tendon does not identify a set of patients that requires operative management for resolution. Our data seem to support this observation as two of seven cases from our cohort did not undergo an operation or I&D despite the presence of multiple Kanavel signs and fluid visualized on POCUS. Neither of these patients was known to have a bad outcome at a minimum of two-month follow-up.

Non-surgical management of suspected flexor tenosynovitis has been previously reported. Nevasier and Gunther reported the successful nonsurgical management of PFT in four patients with a regimen of intravenous antibiotics, splinting, and elevation. The authors noted that all four of these patients presented within 48 hours of symptom onset and hypothesized that the early presentation allowed for successful medical management.^12^ Schecter and colleagues reported on six patients with suspected early tenosynovitis who were medically managed. These patients were noted to have fewer of Kanavel’s signs than in the group requiring surgery (2.1 vs 3.6 respectively).^5^

Our data highlight the potential utility of US in the early diagnosis of tenosynovitis. As this is a retrospective case series, with selection bias and without data on the influence of US in decision-making, further conclusions are not warranted.

## LIMITATIONS

Our study is a retrospective series of patients for whom the consulting hand surgeon made a final diagnosis of flexor tenosynovitis and on whom POCUS had been performed by ED attendings. As such, the results of the study are subject to selection bias.

## CONCLUSION

Our study of seven cases, the first case series in the EM literature on this topic, demonstrates that EPs can use POCUS to identify fluid surrounding the tendons in patients presenting with PFT. We also found that some patients presenting without all four of Kanavel’s signs had the US finding of fluid surrounding the tendon and purulence at operation. Furthermore, we found that two patients were managed successfully with non-operative treatment. We conclude that POCUS may assist in the early diagnosis of PFT, possibly resulting in the success of conservative, non-operative management. We recommend that future study be directed at additional findings of tendon diameter and hyperemic flow in an effort to make POCUS of suspected PFT more specific for the diagnosis.

Documented patient informed consent and/or Institutional Review Board approval has been obtained and filed for publication of this case report.

## Figures and Tables

**Table:** Number of Kanavel signs and history of puncture trauma to the hand, key ultrasound images, treatment course, and final discharge diagnosis.

Case number US machine transducer conduction medium	Number of Kanavel’s signs on ED exam: Previous trauma?	Key image	Operative findings and treatment course	Final discharge diagnosis
Case 1 Sonosite (Seattle, WA) Linear 25mm, 10-5 MHz Gel mattress	4/4 No history of trauma	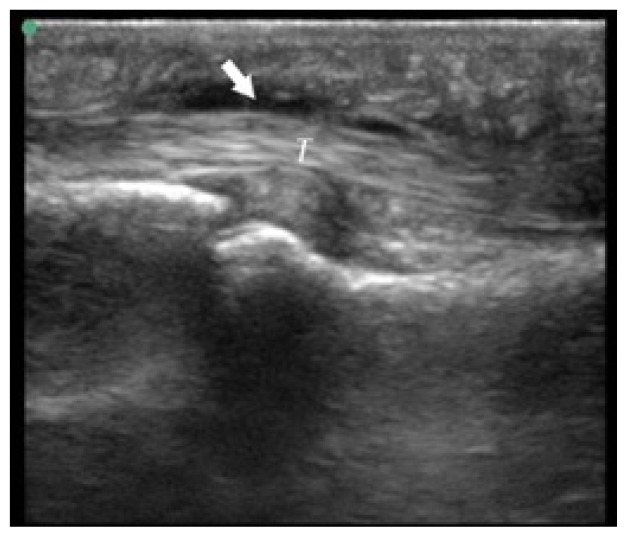	No fluid at operation, Received antibiotics for three days prior to operation	Flexor tenosynovitis
Case 2 Sonosite (Seattle, WA) Linear 25mm, 10-5 MHz Gel mattress	2/4 No history of trauma	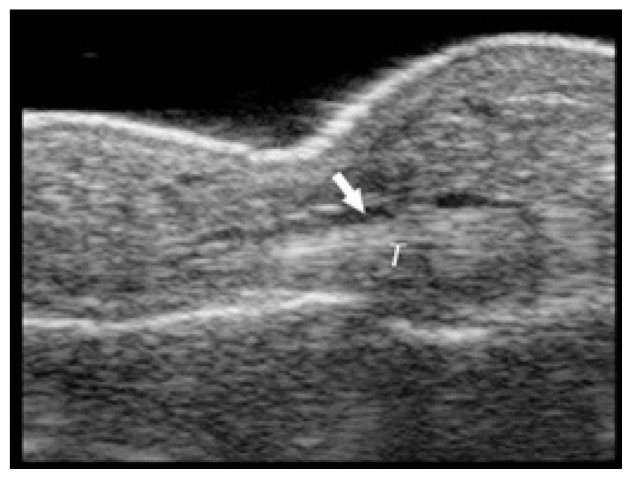	Non-operative management with antibiotics	Flexor tenosynovitis
Case 3 Toshiba (Tokyo, Japan) Linear 32mm, 11-6 MHz Water bath	4/4 History of puncture trauma	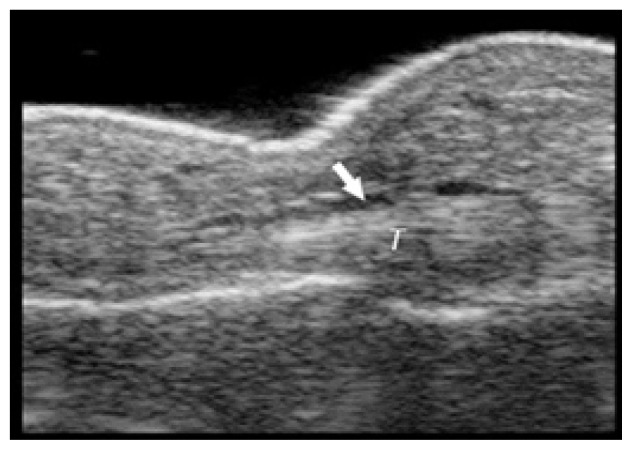	+Purulence and foreign body at operation	Flexor tenosynovitis
Case 4 Sonosite (Seattle, WA) Linear 25mm, 10-5 MHz Gel mattress	2/4 History of puncture trauma	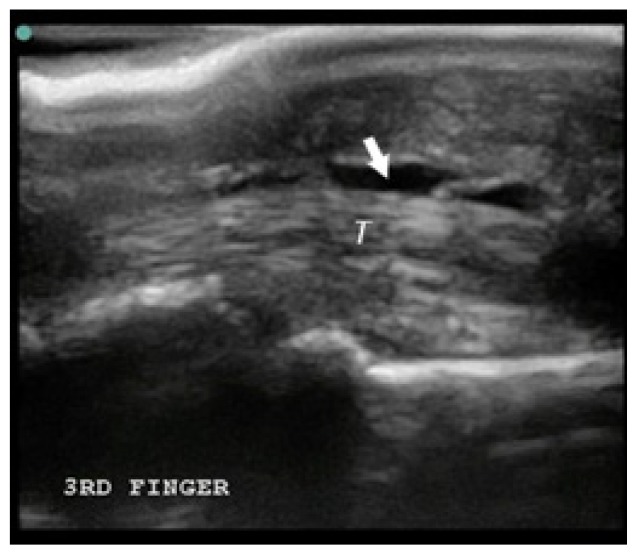	I&D at bedside and IV antibiotics	Possible flexor tenosynovitis
Case 5 Ultrasonix (Vancouver, Canada) Linear 38mm, 14-5 MHz Water bath	4/4 History of puncture trauma	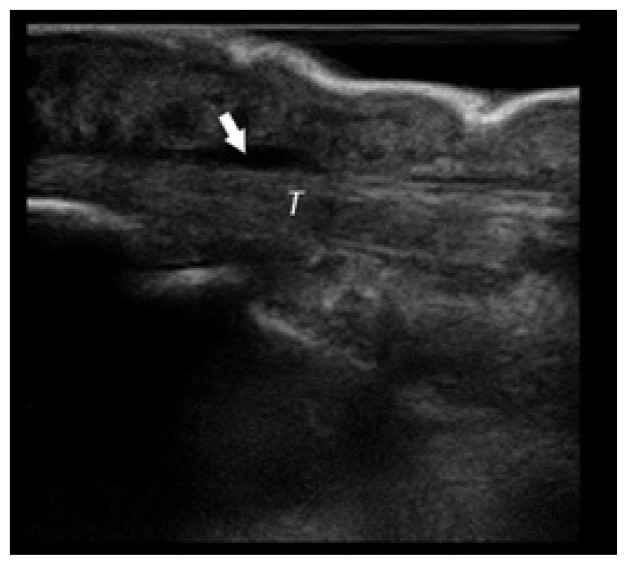	+purulence at operation	Flexor tenosynovitis
Case 6 Sonosite (Seattle, WA) Linear 25mm, 10-5 MHz Gel mattress	3/4 History of puncture trauma	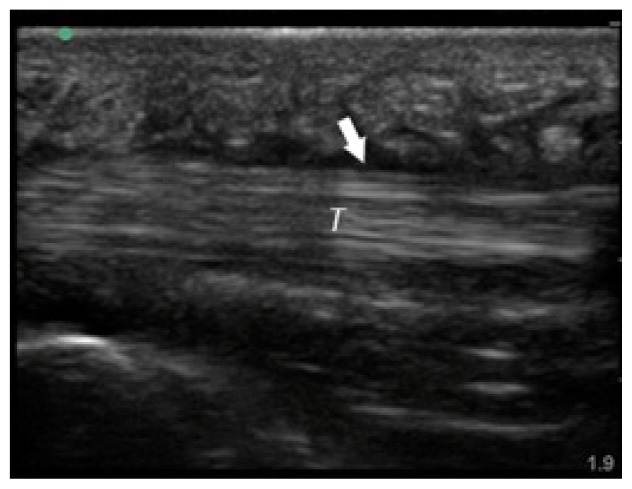	Non-operative management with antibiotics	Flexor tenosynovitis
Case 7 Sonosite (Seattle, WA) Linear 25mm, 10-5 MHz Gel mattress	2/4 History of puncture trauma	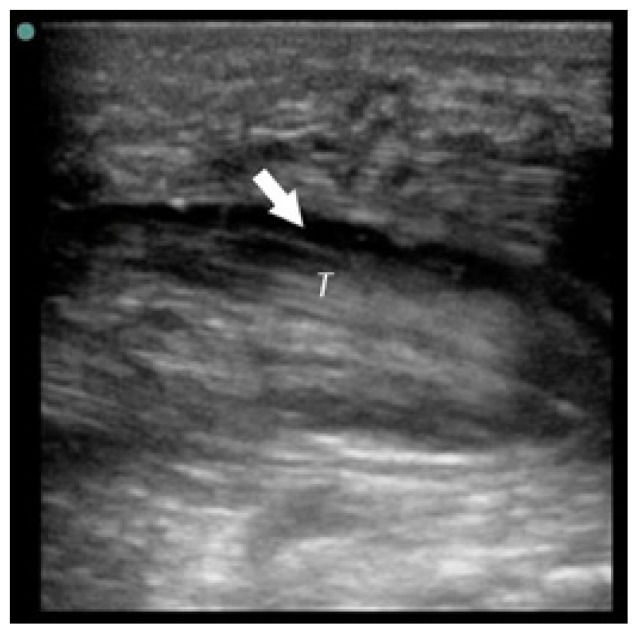	+Purulence at operation	Flexor tenosynovitis

*US,* ultrasound; *ED,* emergency department; *OR*, operating room; *IV*, intravenous, *I & D*, incision and drainage.
